# Cyclic di-GMP signalling and the regulation of bacterial virulence

**DOI:** 10.1099/mic.0.068189-0

**Published:** 2013-07

**Authors:** Robert P. Ryan

**Affiliations:** Division of Molecular Microbiology, College of Life Sciences, University of Dundee, Dundee, UK

## Abstract

Signal transduction pathways involving the second messenger cyclic di-GMP [bis-(3′-5′)-cyclic di-guanosine monophosphate] occur widely in bacteria where they act to link perception of environmental or intracellular cues and signals to specific alterations in cellular function. Such alterations can contribute to bacterial lifestyle transitions including biofilm formation and virulence. The cellular levels of the nucleotide are controlled through the opposing activities of diguanylate cyclases (DGCs) and phosphodiesterases (PDEs). The GGDEF domain of DGCs catalyses the synthesis of cyclic di-GMP from GTP, whereas EAL or HD-GYP domains in different classes of PDE catalyse cyclic di-GMP degradation to pGpG and GMP. We are now beginning to understand how alterations in cyclic di-GMP exert a regulatory action through binding to diverse receptors or effectors that include a small ‘adaptor’ protein domain called PilZ, transcription factors and riboswitches. The regulatory action of enzymically active cyclic di-GMP signalling proteins is, however, not restricted to an influence on the level of nucleotide. Here, I will discuss our recent findings that highlight the role that protein–protein interactions involving these signalling proteins have in regulating functions that contribute to bacterial virulence.

## Introduction

**Fig. 1.**
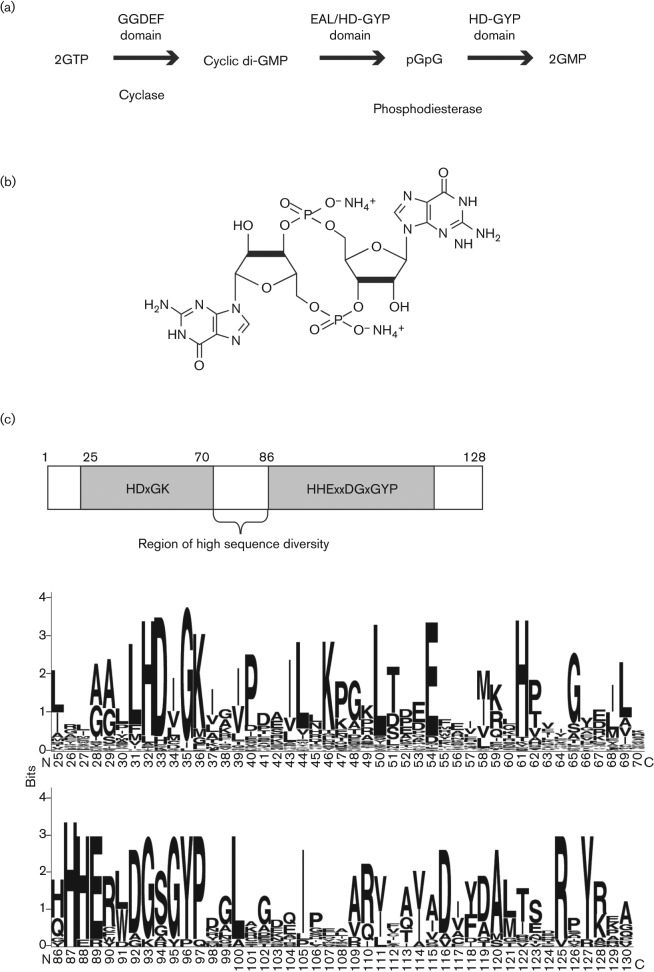
Cyclic di-GMP synthesis and degradation, and the structure of the HD-GYP domain. (a) The role of GGDEF, EAL and HD-GYP domains in the synthesis and degradation of cyclic di-GMP. Synthesis of cyclic di-GMP from two molecules of GTP is catalysed by the GGDEF domain and is predicted to occur in two steps, with pppGpG as an intermediate. Each step releases a molecule of inorganic pyrophosphate. The degradation of cyclic di-GMP to GMP also occurs via a two-step reaction, with the linear dinucleotide pGpG as an intermediate. EAL domains characterized thus far catalyse the first step more efficiently than the second, so that the major product is pGpG, whereas the HD-GYP domain catalyses both steps to yield GMP. Other PDE enzymes, perhaps non-specific, may also convert pGpG to 5′ GMP. (b) Chemical structure of the second messenger cyclic di-GMP. (c) Conservation of amino acid residues and motifs within the HD-GYP domain. Alignments of 251 HD-GYP domains encoded in complete microbial genomes (www.ncbi.nlm.nih.gov/Complete_Genomes/RR census.html) were used to establish conserved residues and the output was drawn using the WebLogo program (weblogo.berkeley.edu). The letters in each position represent amino acid residues found in that position; the height of each letter reflects the fraction of sequences with the corresponding amino acid residue in that position. Positions 71–85 (which are not shown) represent a region of high sequence diversity.

Cyclic di-GMP [bis-(3′-5′)-cyclic di-guanosine monophosphate] was originally described in 1987 as an allosteric regulator of cellulose synthesis in *Acetobacter xylinum* (now *Gluconacetobacter xylinus*) (Fig. 1) (Ross *et al.*, 1987). Cyclic di-GMP signalling is now implicated in regulation in a very wide range of bacteria where it acts to influence many aspects of behaviour, including adhesion to surfaces, aggregation and biofilm formation, developmental transitions and, importantly, the virulence of bacterial pathogens of both animals and plants. The cellular level of cyclic di-GMP results from a balance between synthesis and degradation. Three protein domains are implicated in these processes: the GGDEF domain catalyses synthesis of cyclic di-GMP from two molecules of GTP, whereas EAL and HD-GYP domains catalyse hydrolysis of cyclic di-GMP, firstly to the linear nucleotide pGpG and then at different rates to GMP (Fig. 1) (Paul *et al.*, 2004; Christen *et al.*, 2005; Tischler & Camilli 2004; Ryan *et al.*, 2006). All of these domains are named after conserved amino acid motifs. Most proteins with GGDEF/EAL/HD-GYP domains contain additional signal input domains, suggesting that their activities are responsive to signals or cues from the bacterial cell or its environment.

**Fig. 2.**
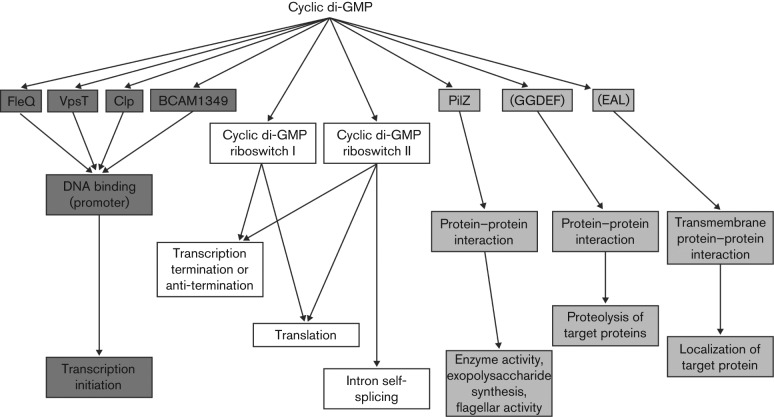
A diversity of receptors/effectors for cyclic di-GMP act to mediate the regulation of multiple cellular processes by the second messenger. Cyclic di-GMP exerts regulation at the level of transcription by binding to different classes of transcription factor. Cyclic di-GMP-controlled riboswitches regulate transcriptional termination or anti-termination or translational initiation. Activity, localization or proteolysis of target proteins, or activities of larger cellular structures, are controlled by cyclic di-GMP-binding effector proteins that act by direct interaction with their targets.

Cyclic di-GMP exerts a regulatory action through binding to diverse receptors or effectors that include small ‘adaptor’ protein domains called PilZ, transcription factors and riboswitches (Fig. 2). Thus, regulation can occur at the level of transcription, post-transcription or post-translation, such as in the allosteric effect on cellulose synthesis or regulation of protein turnover (Fig. 2) (Sondermann *et al.*, 2012; Ryan *et al.*, 2012b). In addition to effects exerted through enzymic modulation of the level of cyclic di-GMP, regulation by enzymically inactive GGDEF, EAL or HD-GYP domain proteins mediated by protein–protein interactions have been described. The range of receptors and the multiplicity of GGDEF, EAL and HD-GYP proteins within the same bacterial cell indicate that there is considerable complexity in the organization of cyclic di-GMP signalling, which is relatively poorly understood. The reader is directed towards several excellent recent reviews of this area (Hengge, 2009; Schirmer & Jenal, 2009; Boyd & O**’**Toole, 2012; Römling *et al.*, 2013).

In the plant pathogen *Xanthomonas campestris* pv. *campestris* (*Xcc*), a two-component system comprising the sensor kinase RpfC and HD-GYP domain regulator RpfG is implicated in perception and signal transduction of the cell-to-cell signal DSF (for diffusible signalling factor) (Barber *et al.*, 1997; Slater *et al*., 2000; Ryan *et al*., 2006, 2009). This Rpf/DSF system regulates the synthesis of virulence factors, including extracellular enzymes and the extracellular polysaccharide xanthan, and influences biofilm formation, pilus-dependent motility and virulence (Slater *et al*., 2000; Ryan *et al*., 2006, 2007). Our studies of RpfG provided the first demonstration of the role of the HD-GYP domain as a phosphodiesterase (PDE) (Ryan *et al.*, 2006). More recent work has shown that RpfG regulates synthesis of the different factors under the control of the Rpf/DSF system by different mechanisms. Regulation of the synthesis of extracellular enzymes, such as endoglucanase, involves the cyclic di-GMP responsive transcriptional regulator Clp (Chin *et al.*, 2010). In contrast, physical interaction of RpfG with certain GGDEF domain-containing proteins is needed to control pilus-dependent motility. These latter protein–protein interactions are dynamic, being promoted by DSF signalling (Andrade *et al.*, 2006; Ryan *et al*., 2010, 2012). Intriguingly, the regulatory action of these GGDEF domain proteins on motility is not dependent on their action in cyclic di-GMP synthesis (Ryan *et al.*, 2012a). These observations support the idea that proteins involved in cyclic di-GMP signalling can be multifunctional, with some activities independent of known enzymic function.

Herein my group’s findings from studies of the Rpf/DSF system in *Xcc* and their broader relevance to an understanding of regulation of virulence by cyclic di-GMP in other pathogens are discussed. This begins with a brief overview of the DSF signalling system in *Xcc*, before going on to review in more detail what is known of the multiple regulatory functions of RpfG and the HD-GYP domain, how such diverse regulatory actions are exerted and the bifunctional nature of certain GGDEF domain proteins.

## The HD-GYP domain protein RpfG links cell-to-cell signalling to pathogenesis in *Xcc*

### The rpfBFCG gene cluster of *Xcc* encodes a cell–cell signalling system

The *rpf* gene cluster (for regulation of pathogenicity factors) was identified originally as being required for full virulence in *Xcc* (Tang *et al*., 1991; Slater *et al.*, 2000). This cluster consists of nine genes (*rpfA*–*I*) that are located within a 21.9 kb region of the chromosome of *Xcc*. Mutation of *rpfF*, *rpfG* and *rpfC* genes leads to a coordinate downregulation of the synthesis of a number of extracellular enzymes, including endoglucanase, protease, endomannanase and polygalacturonate lyase, and of the extracellular polysaccharide xanthan, but an increase in biofilm formation (Slater *et al.*, 2000).

**Fig. 3.**
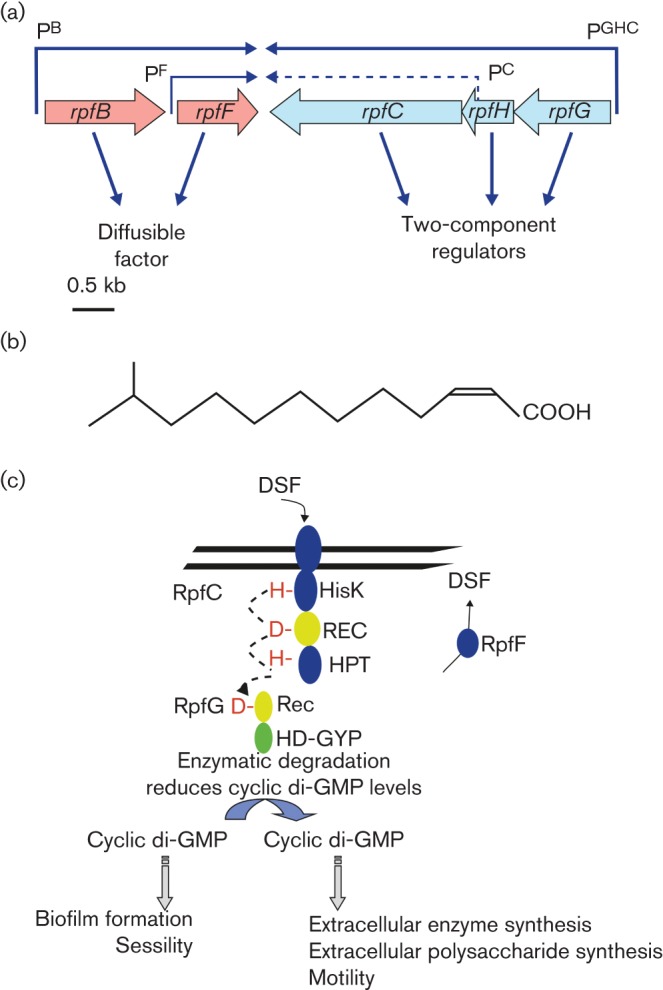
The role of Rpf proteins in the synthesis, perception and signal transduction of the diffusible signal factor (DSF) in *Xcc*. (a) Physical map and transcriptional organization of the part of the *rpf* gene cluster of *Xcc* from *rpfB* to *rpfG* involved in DSF signalling. The organization of ORFs predicted by sequence analysis together with predicted directions of transcription are indicated by the broad arrows. The positions of the experimentally determined transcriptional start sites together with the predicted transcripts are indicated by arrows above the ORFs: *rpfG*, *rpfH* and *rpfC* are transcribed as an operon from a promoter P^GHC^, upstream of *rpfG*; *rpfC* has its own (weak) promoter, P^C^, located within *rpfH*, this is indicated by the dashed arrow; *rpfB* and *rpfF* are transcribed as an operon from a promoter upstream of *rpfB*, P^B^, although *rpfF* has its own promoter, P^F^. RpfF and RpfB are implicated in the synthesis of the DSF; RpfG and RpfC compose a two-component regulatory system. RpfH encodes a protein with amino acid sequence similarity to the sensory input domain of RpfC, but has no apparent regulatory role. (b) Chemical structure of the signal molecule DSF (*cis*-11-methyl- dodecenoic acid) of *Xcc*. (c) Model of DSF signalling in *Xcc.* The synthesis of the DSF signal requires RpfF and is partially dependent on RpfB. DSF perception and signal transduction involve the complex sensor RpfC and HD-GYP domain regulator RpfG. Autophosphorylation of RpfC occurs in response to ligand (DSF) binding. This is followed by phosphorelay and phosphotransfer to the regulator RpfG (broken arrow). Phosphorylation of RpfG leads to its activation as a cyclic di-GMP PDE, an activity associated with the HD-GYP domain. The consequent alterations in the level of cyclic di-GMP affect the synthesis of virulence factors and biofilm dispersal. REC, two-component receiver; HPT, histidine phosphotransfer; HisK, histidine kinase phosphoacceptor.

RpfF and RpfB direct the production of the DSF signal molecule, which has been characterized as the unsaturated fatty acid *cis*-11-methyl-dodecenoic acid (Fig. 3) (Barber *et al.*, 1997; Slater *et al.*, 2000; Dow *et al.*, 2003; Wang *et al.*, 2004). Synthesis of DSF is completely dependent on RpfF, which belongs to the crotonase superfamily of enzymes, but is only partially dependent on RpfB, which has similarity to a long chain fatty acyl-CoA ligase. The *rpfB* and *rpfF* genes are co-transcribed from a promoter upstream of *rpfB*, although *rpfF* also has its own promoter (Slater *et al.*, 2000). Addition of DSF to *rpfF* mutants, but not to *rpfB* mutants, can phenotypically restore the production of extracellular enzymes and xanthan and cause the dispersal of aggregates (Barber *et al.*, 1997; Vojnov *et al.*, 2001; Dow *et al.*, 2003). Although DSF is synthesized within the bacterial cell, it is believed that it is free to diffuse out of the cell because of its lipophilic nature. In the extracellular medium the levels of DSF could be influenced by a number of external factors, including the volume of a microenvironment or the rate of flow through an open environment such as the vascular system of a plant host which is colonized by *Xcc* during black rot disease. In this way DSF may act as a sensor for confinement and not just a monitor of the size of the population of producing cells.

### DSF perception and transduction via the two-component system RpfC and RpfG

The two-component system made up of RpfC and RpfG is encoded within the *rpfGHC* operon and convergently transcribed to the adjacent *rpfF* gene (Fig. 3) (Slater *et al.*, 2000). RpfC is a multidomain sensory histidine kinase comprising sensory input, histidine kinase, CheY-like response regulator and C-terminal histidine phosphotransfer (Hpt) domains. RpfG is an unusual regulator in that it contains a CheY-like regulatory input domain (REC domain) fused to an HD-GYP domain, the function of which is discussed in detail below (Slater *et al.*, 2000; Ryan *et al.*, 2006). RpfH has some sequence similarity to the sensory input domain of RpfC, but has no predicted phosphorylatable histidine residue and no apparent regulatory role.

Several lines of evidence implicate the RpfC/RpfG system in the perception and transduction of the DSF signal. Strains lacking RpfC or RpfG are ‘deaf’ to the addition of DSF, which cannot restore extracellular enzyme synthesis or induce aggregate dispersal in *rpfG* and *rpfC* mutants (as it does in *rpfF* mutants) (Slater *et al.*, 2000; Dow *et al.*, 2003; Ryan *et al.*, 2006). In addition, the RpfC/RpfG two-component system has been reconstructed in *Pseudomonas aeruginosa* and shown to confer responsiveness to exogenously added DSF, as seen through effects on swarming motility. These findings support a model for DSF signal transduction in which recognition of DSF leads to autophosphorylation of the sensor RpfC and phosphotransfer to RpfG, thereby activating it for regulation (Fig. 3) (Ryan *et al.*, 2006). Although the phenotypes of *rpfF*, *rpfC* and *rpfG* mutants for virulence, synthesis of certain virulence factors such as extracellular enzymes and biofilm formation are very similar, the possibility that DSF is also recognized by other sensors in *Xcc* or that RpfC recognizes other environmental cues in addition to DSF cannot be excluded. Recent evidence to support this view is discussed later in this article.

### Potential dual role of the DSF sensor kinase RpfC

As well as being implicated in the perception of DSF and signal transduction leading to virulence factor synthesis, RpfC is also involved in the regulation of DSF synthesis (He *et al.*, 2006; Cheng *et al.*, 2010). Mutation of *rpfC* leads to an increase in the level of DSF by up to 15-fold over the wild-type level. This led to the suggestion that RpfC participates in a negative feedback loop that serves to regulate DSF levels (He *et al.*, 2006; Cheng *et al.*, 2010). Although conserved amino acid residues of RpfC are implicated in phosphorelay and essential for activation of production of extracellular enzymes and xanthan, they are not required for this second role of RpfC in repression of DSF biosynthesis (He *et al.*, 2006; Cheng *et al.*, 2010). The available evidence suggests that this latter effect is mediated instead by protein–protein interactions between the REC domain of RpfC and RpfF, the DSF synthase. Sequestration of RpfF in this manner may restrict DSF synthesis. Release of RpfF as a result of conformational changes in RpfC that occur upon DSF binding may act as a mechanism to allow rapid autoinduction of DSF synthesis. Thus, RpfC has a dual signalling function, with phosphorelay to control synthesis of extracellular enzymes, and EPS and protein–protein interaction to control DSF synthesis. This latter mechanism represents an added dimension to conventional two-component signalling paradigms. However, it remains to be seen whether such interactions can be detected in *Xcc* expressing wild-type levels of the proteins.

## The HD-GYP domain of RpfG is a cyclic di-GMP PDE

The suggestion that the HD-GYP domain was a second class of PDE involved in cyclic di-GMP signalling came originally from bioinformatic studies (Galperin *et al.*, 1999; 2001). HD-GYP is a subgroup of the HD superfamily of metal-dependent phosphohydrolases. The association of this domain with REC domains in many bacterial proteomes indicated a role in signalling. Analysis of the distribution and numbers of GGDEF, EAL and HD-GYP domains encoded in various bacterial genomes was the basis for the suggestion that the HD-GYP domain was involved in cyclic di-GMP signalling. A consensus sequence of the HD-GYP domain from alignment of more than 200 proteins indicated that the signature HD and GYP motifs might be more usefully considered as part of the larger motifs HDxGK and HHExxDGxGYP, which are present in subdomains separated by a region of high sequence diversity (Fig. 1) (Ryan *et al*., 2010). In addition, there are a number of other well-conserved charged and hydrophobic residues (Ryan *et al*., 2010). It has been proposed that other conserved H and D residues (i.e. those not in the HD diad) have a role in metal binding in these metal-dependent enzymes, and that the GYP motif has a role in determining substrate specificity, but currently there is no experimental evidence to support this assertion.

The role of RpfG as a cyclic di-GMP PDE in *Xcc* was initially indicated by genetic experiments and the demonstration of the enzymic activity of the isolated HD-GYP domain (Ryan *et al.*, 2006). Indirect evidence of the role of RpfG in cyclic di-GMP regulation came from observations that expression of genes encoding the EAL domain (with PDE activity) in the *rpfG* mutant of *Xcc* restores extracellular enzyme and xanthan synthesis towards wild-type levels and triggers biofilm dispersal (Ryan *et al.*, 2006). Conversely, expression of a gene encoding a GGDEF domain protein [with diguanylate cyclase (DGC) activity] in wild-type *Xcc* causes biofilm formation and represses the synthesis of extracellular enzymes, thus giving a phenotype identical to an *rpfG* mutant (Ryan *et al.*, 2006).

The description of the biochemical function of RpfG was greatly facilitated by the finding that the isolated HD-GYP domain is able to partially restore extracellular enzyme synthesis to *rpfG* mutants of *Xcc* (Ryan *et al.*, 2006). The HD-GYP domain was isolated as a His-tagged protein and shown to have metal-dependent cyclic di-GMP PDE activity, but no activity against GTP, GMP or cyclic mononucleotides (Ryan *et al.*, 2006). The final product of cyclic di-GMP degradation was GMP, although there was evidence that pGpG was an intermediate product. Importantly, it was shown that this enzymic activity of the HD-GYP domain is required for its regulatory activity.

Site-directed mutagenesis to alter the H and D residues that form the presumed catalytic diad to A residues abolished both the regulatory activity in restoration of extracellular enzyme synthesis and the enzymic activity against cyclic di-GMP (Ryan *et al.*, 2006). The HD-GYP domain, but not intact RpfG, can also restore extracellular enzyme synthesis to the *rpfGHC* mutant (a strain that does not have a sensor kinase RpfC) (Ryan *et al.*, 2006). This suggests that in the absence of activation by phosphorylation from RpfC, the receiver domain acts to inhibit the cyclic di-GMP PDE activity of the HD-GYP domain.

In this proposed model, sensing of DSF would lead to phosphorylation of the REC domain, thus relieving this inhibition, thereby leading to a reduction in the level of cyclic di-GMP. Accordingly, mutation of *rpfF,* which leads to the absence of DSF, and mutation of *rpfG* both lead to elevation of the cellular level of cyclic di-GMP (Ryan *et al.*, 2010; R.P Ryan and J.M. Dow).

## RpfG influences extracellular enzyme synthesis and biofilm formation by means of cyclic di-GMP turnover

Mutation of *rpfG* in *Xcc* leads to a reduction in the synthesis of extracellular enzymes and an aggregated growth behaviour when grown in certain media (Ryan *et al.*, 2006), effects that are associated with an elevated level of cyclic di-GMP. These observations are consonant with work on cyclic di-GMP signalling in diverse bacteria that supports a general principle that higher cellular levels of the nucleotide promote biofilm formation and sessility, whereas lower cellular levels promote the synthesis of extracellular enzymes and motility (Ryan *et al.*, 2006). In *Xcc*, some of the pathways and effectors that link altered levels of the nucleotide in the cell to these different phenotypes of altered extracellular enzyme synthesis and formation of aggregates or biofilms have been described.

### Clp is a cyclic di-GMP receptor that regulates expression of specific genes

Recent findings in *Xcc* have implicated the cyclic-AMP receptor-like protein Clp as an element responsible for linking Rpf/DSF signalling (and alteration in cyclic di-GMP) to the expression of genes encoding extracellular enzymes (Leduc & Roberts, 2009; Tao *et al.*, 2010; Chin *et al.*, 2010). The Clp transcription factor is so-named as it exhibits high sequence similarity to the cAMP receptor protein (CRP) of *Escherichia coli*. Both proteins have an N-terminal cyclic mononucleotide- (cNMP-) binding domain and a C-terminal DNA-binding domain (Leduc & Roberts, 2009; Tao *et al.*, 2010; Chin *et al.*, 2010). CRP of *E. coli* is well established as a regulator of carbon source utilization. In contrast, Clp of *Xcc* does not affect the utilization of carbon sources, but significantly alters the expression of a number of virulence genes, including those encoding extracellular enzymes such as the endoglucanase EngXCA and the endomannanase ManA. Although CRP binding to target promoters requires cyclic AMP, Clp binds to the promoter of *engXCA* and other target genes in the absence of any nucleotide. The crystal structure of Clp shows that the protein has an intrinsic conformation adapted for DNA binding in the absence of any ligand (Chin *et al.*, 2010). Clp binds cyclic di-GMP with an affinity that is physiologically relevant, and this binding prevents the binding of Clp to target promoters (Chin *et al.*, 2010). The binding site for cyclic di-GMP in Clp is not known, although modelling studies suggest that the nucleotide binds at a site between the cNMP- and DNA-binding domains, rather than at the cNMP-binding domain (Chin *et al.*, 2010).

As outlined above, the Rpf/DSF system negatively influences biofilm formation, as well as positively regulating the synthesis of extracellular enzymes and motility (Ryan *et al.*, 2006, 2007). Several lines of evidence suggest that Clp plays a role in the regulation of biofilm dynamics in response to alterations in the cyclic di-GMP level. Mutation of *clp* leads to the downregulation of expression of *manA* that encodes the endomannanase ManA, which is implicated in biofilm dispersal and, conversely, in the upregulation of *xag* gene expression, which is implicated in biofilm formation (Lu *et al.*, 2012). The binding of Clp to promoters of both *manA* and *xag* genes is inhibited by cyclic di-GMP. A parsimonious explanation for these findings is that Clp can act both as an activator and a repressor of transcription of different genes (Lu *et al.*, 2012). However, the evidence provided does not rule out the possibility that regulation of *xag* gene expression by Clp is more indirect, for example by positive regulation of a distinct repressor protein. These findings suggest that Clp links elevated levels of cyclic di-GMP to the altered expression of two factors that are known to influence biofilm formation. However, mutation of *clp* alone does not influence biofilm formation, indicating the existence of additional cyclic di-GMP-regulated functions or pathways that influence this developmental transition.

### High-resolution transcriptional analysis reveals the extent of the regulatory influence of RpfG on virulence-related functions

**Fig. 4.**
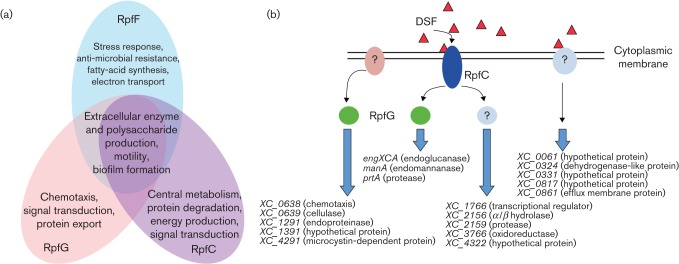
Global transcriptional analysis reveals DSF/Rpf signalling as a network rather than a linear pathway. (a) Overlapping and discrete regulatory actions of RpfF, RpfG and RpfC on functions that are involved in phytopathogenesis as revealed by RNA-Seq and directed mutagenesis. (b) Depiction of a regulatory network with pathways involving DSF, RpfC and RpfG in the control of virulence factor synthesis in *Xcc.* Expression of several genes including *engXCA, prtA* and *manA* is co-ordinately regulated by RpfF, which synthesizes DSF and the RpfCG two-component system, consistent with the linear pathway previously described. However, RpfG controls a number of genes including *XC_0638* (predicted to encode a chemotaxis protein) that are not influenced by RpfF or RpfC, suggesting an interaction of RpfG with a second unknown sensor (left). DSF and RpfC also regulate expression of a number of genes including *XC_1766* (predicted to encode a transcription regulator) in a pathway independent of RpfG (centre). Finally, several genes including *XC_0061* (encoding a hypothetical protein) are regulated by DSF but not by RpfG or RpfC, suggesting the occurrence of a second sensor and signal transduction system for DSF (right). All of the target genes indicated encode virulence factors that are novel, with the exception of *engXCA*, *prtA* and *manA,* whose role in virulence has been described previously. All predicted protein functions are given in parentheses.

Although the significance of the Rpf/DSF signalling system for the virulence of *Xcc* is now well established, much remains to be understood about the action of the signalling system and the underlying downstream regulatory mechanisms. A recent approach to address this gap in understanding used RNA-Seq to define the regulatory influence of different Rpf proteins on the transcriptome of *Xcc* (An *et al.*, 2013). The findings supported previous observations by the demonstration that mutation of *rpfF*, *rpfC* and *rpfG* all give rise to reduced transcript levels for genes encoding endoglucanase, various proteases and endomannanase (Slater *et al.*, 2000; Ryan *et al.*, 2007). Nevertheless, the genes influenced co-ordinately by *rpfF*, *rpfC* and *rpfG* only correspond to a small proportion (~2 %) of those whose transcript level is significantly altered. Contrary to expectation, the regulatory effects of RpfC and RpfG on transcript levels have limited overlap. Furthermore, mutations of *rpfG* and *rpfC* have divergent effects on the transcript levels of a number of genes (Fig. 4). These observations suggest that the Rpf/DSF system in *Xcc* is considerably more complex than previously thought, and point to the existence of other regulators that interact with RpfC and of other sensors that interact with RpfG. However, the observation that all of the genes whose transcript levels are commonly influenced by RpfF and RpfG are under the influence of RpfC suggests that such alternative sensors do not recognize DSF (Fig. 4). It is important to note that these experiments were carried out in complex media so analysis under further growth conditions may identify further complexity of regulation.

Overall, this analysis allowed the identification of many genes that were previously undetected by computational analysis of the genome sequence and novel transcribed regions of the genome, such as potential non-coding RNA genes, that are under RpfG control. Importantly mutational studies allowed the description of over 60 new virulence factors within the group of RpfG-controlled genes. Further experimentation is now needed to address the molecular basis of RpfG regulation of expression of the encoding genes.

## RpfG exerts regulatory effects that are independent of cyclic di-GMP

### Protein–protein interactions and the regulation of specific phenotypes

As discussed above, one role for RpfG in DSF signal transduction is as a PDE, modulating the level of cyclic di-GMP to influence expression of certain virulence genes, such as those encoding extracellular enzymes. However, RpfG also acts in a distinct pathway to regulate a different DSF/Rpf-regulated virulence function, that of pilus-dependent motility (Ryan *et al*., 2010, [Bibr r27]2012). This distinct regulatory effect is mediated by protein–protein interactions with specific GGDEF domain (DGC) proteins. The initial indication of this role for RpfG came from a yeast two-hybrid (Y2H) screen that showed that the HD-GYP domain of RpfG of the citrus canker pathogen *Xanthomonas axonopodis* pv. *citri* interacts with a subset of GGDEF domain-containing proteins (Andrade *et al.*, 2006). The occurrence of these interactions in *Xcc* was then directly examined by fluorescence resonance energy transfer (FRET) analysis (Ryan *et al*., 2010). The findings indicated that physical interactions between RpfG and the GGDEF domain proteins XC_0249 and XC_0420 occur within the *Xcc* cell under the growth conditions used (Ryan *et al*., 2010). Furthermore, these interactions between RpfG with XC_0249 and XC_0420 are dynamic and are promoted during DSF signalling. This is correlated with the relocalization of RpfG to the cell poles, where the DSF sensor kinase RpfC is located (Ryan *et al*., 2010). It has been proposed that this localization of RpfG in response to DSF is associated with the sensing of the signal by RpfC, autophosphorylation of the sensor and subsequent phospho-transfer to the REC domain of RpfG. In this view, the un-phosphorylated REC domain negatively influences the ability of the HD-GYP domain to interact with GGDEF domain proteins (as well as the enzymic activity against cyclic di-GMP). Sensing of DSF and phosphorylation of the REC domain relieves this inhibition, promoting the physical interaction with the GGDEF domain proteins.

### The role of the GYP and DxD motifs in HD-GYP : : GGDEF protein interaction

The strategy to examine the biological role of HD-GYP : : GGDEF domain interactions in *Xcc* was to identify residues or motifs critical for interaction using alanine-substitution mutagenesis within the HD-GYP domain and then to examine the phenotypic consequences of such changes. Mutagenesis studies coupled with Y2H and FRET in *Xcc* established that the GYP motif within the HD-GYP domain is required for interaction with XC_0249 and XC_0420 (Ryan *et al*., 2010, 2012a). Alanine substitutions within the GYP motif of RpfG have no effect on expression of some RpfG-regulated phenotypes such as the synthesis of extracellular enzymes and biofilm formation, but do influence pilus-dependent motility. In accordance with these findings, double mutation of *XC_0249* and *XC_0420* (but not individual mutation) leads to a reduction in motility, but has no effect on the synthesis of extracellular enzymes or biofilm formation.

A parallel approach using synthetic peptide overlay technology (SPOT) in conjunction with alanine-substitution mutagenesis allowed the identification of the DxD motif within the GGDEF domain proteins as being required for inter-domain interaction (Ryan *et al.*, 2012a). The role of this motif for interaction and for regulation of motility was confirmed by FRET and phenotypic analysis in *Xcc*. The DxD motif is distinct from the catalytic A site (GGD/EEF) or the regulatory I site and is conserved in a number of GGDEF domain proteins, suggesting that GGDEF : : HD-GYP interactions may be similarly widespread.

Both of the target GGDEF domain proteins are active as DGCs *in vitro*. Surprisingly however, further mutational analysis demonstrated that the GGDEF domain proteins do not depend upon their DGC activity to regulate motility (Ryan *et al.*, 2012a). This is indicative of a potential bifunctional nature of these cyclic di-GMP signalling proteins.

### Complex formation between HD-GYP, GGDEF and PilZ domain proteins regulates motility in *Xcc*

In order to uncover the mechanism by which HD-GYP : : GGDEF inter-domain interactions specifically regulate pilus-dependent motility, Y2H analysis was employed with the GGDEF domains from XC_0249 and XC_0420 as baits. These screens, together with far-Western analysis, identified that the PilZ domain protein XC_2249 interacted with both GGDEF domains providing indirect evidence that interactions between the domains HD-GYP/GGDEF/PilZ occur within *Xcc*. Intriguingly, XC_2249 had been previously implicated in regulation of pilus-dependent motility and virulence in *Xcc* (McCarthy *et al*., 2008).

To provide more direct evidence of multi-protein complexes forming *in vivo* between RpfG, GGDEF domain proteins and the PilZ domain protein XC_2249 within *Xcc*, a tri-chromophore FRET approach was used (Ryan *et al.*, 2012a). These data suggest that a tri-protein complex of HD-GYP, GGDEF and PilZ domain forms. Although the PilZ domain protein XC_2249 is able to bind cyclic di-GMP, binding is not required for the regulation of motility or for complex formation with the GGDEF domain proteins.

**Fig. 5.**
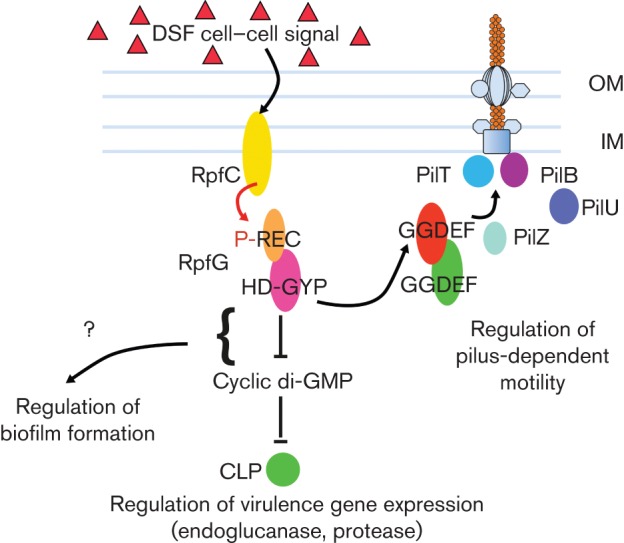
DSF signalling regulates pilus action in *Xcc* by promoting formation of a complex of proteins with HD-GYP, GGDEF and PilZ domains. Surface motility in *Xcc* requires the pilin PilA, the major subunit of the filament of type IV pilus. After being processed by the prepilin peptidase, pilin subunits associated with the inner membrane are incorporated into the base of the pilus filament in a process that requires the inner-membrane protein PilC and the ATPase activity of PilB, which forms a hexameric ring associated with the cytoplasmic face of the inner membrane. The growing pilin polymer passes through pores formed by PilQ in the outer membrane. Pili are depolymerized in a process that requires the hexameric PilT ATPase. DSF perception and signal transduction by the sensor RpfC and HD-GYP domain regulator RpfG can influence pilus function post-translationally; the figure illustrates a model for this process. Autophosphorylation of RpfC in response to ligand (DSF) binding is followed by phosphorelay and phosphotransfer to RpfG. Phosphorylation of RpfG promotes interacts with two GGDEF domain proteins, XC_0249 and XC_0420, an interaction requiring the GYP motif of the HD-GYP domain and DxD motif of the GGDEF domain. This complex goes on to recruit a specific PilZ domain protein (XC_2249) that allows interaction with the PilT (and PilU) pilus motor proteins, thereby modulating pilus-dependent motility. This effect of DSF signalling on motility does not require the enzymic activity of the GGDEF domain proteins, nor cyclic di-GMP binding by the PilZ domain protein XC_2249. OM, outer membrane; IM, inner membrane.

A further Y2H screen with XC_2249 (PilZ) as bait was used to identify possible targets for XC_2249 (PilZ) action (Ryan *et al.*, 2012a). The screen identified the pilus motor proteins PilT and PilU as potential interactors (Ryan *et al.*, 2012a). These proteins are implicated in pilus retraction in xanthomonads and other Gram-negative bacteria. Furthermore, far-Western, tri-chromophore FRET and mutational analysis demonstrate that this XC_2249 (PilZ) protein directly interacts with PilU and PilT, in a manner that depends upon interaction with the GGDEF domain proteins (Ryan *et al.*, 2012a). These data suggest that the interaction of the tri-protein complex of HD-GYP, GGDEF and PilZ domain proteins with PilU and PilT acts to modulate pilus function and as a consequence motility (Fig. 5).

As detailed above, DSF signalling promotes the formation of a complex between the HD-GYP domain response regulator RpfG and two GGDEF domain proteins to regulate pilus-dependent motility (Fig. 5). The interaction of the HD-GYP, GGDEF and PilZ protein complex with PilU and PilT is also controlled by DSF signalling. Overall, these data show that signals can regulate the formation of complexes between proteins with different domains implicated in cyclic di-GMP signalling in a tightly regulated and dynamic fashion. Importantly, such complexes can have an action to regulate bacterial functions in a manner that is independent of the enzymic activity in cyclic di-GMP turnover or binding. It is important to note that cyclic di-GMP does regulate motility in *Xcc*, an effect that is likely to be exerted through an influence on distinct and multiple functions.

These findings contribute to a growing body of work that demonstrates cyclic di-GMP signalling proteins act to regulate different functions in diverse bacteria through participation in protein–protein complexes (Boyd & O**’**Toole, 2012). In some cases, the complexes involve proteins that have GGDEF and/or EAL domains but that are inactive in cyclic di-GMP synthesis or degradation. The recruitment of the enzymically active PdeA to the pole of *Caulobacter crescentus* requires the response regulator CpdR, while the GGDEF domain protein PopA, which lacks DGC activity, recruits the cell cycle regulator CtrA to the pole (Abel *et al.*, 2011). Further examples of complexes involving enzymically inactive proteins include the blue-light-dependent interaction of YcgF (EAL domain) with YcgE, a MerR-like repressor in *E. coli* (Tschowri *et al.*, 2009, 2012), and the interaction of LapD (GGDEF and EAL) with the periplasmic protease LapG in *Pseudomonas fluorescens* (Newell *et al.*, 2011).

## Role of HD-GYP domain proteins in other pathogenic bacteria

Studies of the biological role of HD-GYP domain proteins have been largely restricted to work on *Xcc* and related organisms. However, recent work has addressed the role of these proteins in the unrelated pathogens *P. aeruginosa*, *Vibrio cholerae* and *Borrelia burgdorferi*.

The genome of the human pathogen *P. aeruginosa* PAO1 encodes two proteins (PA4108, PA4781) with an HD-GYP domain and a third protein, PA2572, which contains a variant domain with the sequence YNIGK in place of HDMGK seen in RpfG (Ryan *et al.*, 2010). Heterologous expression of the isolated HD-GYP domains of PA4108 and PA4781 in the *rpfG* mutant of *Xcc* reduces cyclic di-GMP levels. Furthermore, mutation of *PA4108* and *PA4781* leads to an increase in the level of cyclic di-GMP in *P. aeruginosa*. These findings are consistent with the predicted PDE activity of the encoded proteins. In contrast, expression of the variant HD-GYP domain of PA2572 in *Xcc* or mutation of *PA2572* in *P. aeruginosa* has no effect on cyclic di-GMP levels, indicating that the YN-GYP variant domain is enzymically inactive.

Mutation of *PA4108*, *PA4781* and *PA2572* has distinct effects on the biofilm formation and architecture of *P. aeruginosa.* In addition, all three proteins contribute to the virulence of *P. aeruginosa* to larvae of the Greater Wax moth *Galleria mellonella*, although they have differing effects on the production of *P. aeruginosa* virulence factors and on swarming motility. Mutation of either *PA4108* or *PA4781* leads to a reduction in swarming, whereas mutation of *PA2572* has no effect. However, PA2572 has a dominant negative influence on swarming that is cryptic and is revealed only after removal of an uncharacterized C-terminal domain. Complementation of the *PA4108* or *PA4781* mutant with the cognate cloned wild-type gene restores motility and biofilm formation back to wild-type. Mutated alleles expressing variant proteins with a substitution in the HD diad are, however, unable to complement the strains, indicating that the regulatory effects of PA4108 and PA4781 on motility and biofilm formation require their enzymic activity against cyclic di-GMP. The effects on virulence are interesting in the light of previous work that showed that expression of all the three genes is substantially increased during co-culture of *P. aeruginosa* with human epithelial cells, and that *PA2572* and *PA4781* are induced by mucopurulent fluid from cystic fibrosis patients (Ichikawa *et al.*, 2000; Chugani & Greenberg, 2007; Wolfgang *et al.*, 2004).

A number of recent studies have described elements of cyclic di-GMP signalling in the Lyme disease spirochaete, *B. burgdorferi*, and their importance to the lifestyle of the pathogen (Sultan *et al.*, 2010; 2011). The *B. burgdorferi* genome encodes one GGDEF domain DGC (Rrp1) and only two PDEs; PdeA has an EAL domain and PdeB has an HD-GYP domain. Mutational analysis indicates that Rrp1 is essential for survival of the spirochaete in the tick vector, *Ixodes scapularis*, but not for infection of mice, whereas PdeA is required for infectivity in mice. Mutation of *pdeB* leads to a reduced ability to survive in the tick vector and significantly increases flexing, indicating a role for this HD-GYP domain protein in motility. Furthermore, the *in vitro* activity of the recombinant HD-GYP domain protein PdeB as a PDE against cyclic di-GMP has been demonstrated.

Work in *V. cholerae* has addressed the role that HD-GYP domain proteins may have in signal transduction mechanisms associated with quorum sensing (QS) in the El Tor biotype (Hammer & Bassler, 2009). Using *lux* fusions, a number of genes whose expression is upregulated either in response to exogenous autoinducers or in QS mutants that are ‘locked’ in a high cell density mode have been identified (Hammer & Bassler, 2009). It was shown that expression of four genes (*vca0210, vca0681, vca0931* and *vc2340*) that encode HD-GYP domain proteins in *V. cholerae* El Tor is activated by QS. Each of the encoded proteins has the conserved HD and GYP motifs, as well as several other conserved residues, suggesting that they are active as PDEs. In *V. cholerae* El Tor, QS negatively regulates the production of exopolysaccharide and biofilm formation. Accordingly, overexpression of VCA0681 in *V. cholerae* El Tor leads to a reduction in cellular levels of cyclic di-GMP, a decrease in expression of the *vps* gene cluster which directs extracellular polysaccharide synthesis and a reduction in biofilm formation. These regulatory effects are associated with the PDE activity of VCA0681; a variant with a D to A substitution within the HD catalytic diad has little effect on cyclic di-GMP levels and does not influence either *vps* expression or biofilm formation. In contrast to overexpression, mutation of individual genes encoding HD-GYP domain proteins does not lead to any alteration in *vps* expression or biofilm phenotype, suggesting possible redundancy in the action of these proteins.

The findings from these three studies illustrate two aspects of HD-GYP domain proteins that are consonant with our understanding of the diverse roles of other cyclic di-GMP signalling proteins. The first is that different HD-GYP domain proteins appear to have distinct regulatory roles. The second is that although domains with sequences that diverge from the consensus may have lost enzymic activity, they may still play a regulatory role.

## Concluding remarks and future questions

Although identification and characterization of the role of the HD-GYP domain in the regulation of bacterial processes in *Xcc* has improved our knowledge of an element of the ‘core pathway’ of cyclic di-GMP signalling in bacteria, we still have limited understanding of the full role of this domain and the diversity of its action in different bacteria. Although studies of RpfG have revealed some insight into the role of signature (H, D, G, Y, P) residues, the role of other highly conserved residues, such as those within the more extended motif HHExxDGxGYP, is unknown. These residues could conceivably have roles in metal co-factor and substrate binding, enzymic action or in protein–protein interactions with GGDEF domain proteins. High-resolution crystallography has already provided great insights into the mechanisms of action of GGDEF and EAL domain proteins and various cyclic di-GMP effectors (Schirmer & Jenal, 2009). However, only limited information on the HD-GYP domain is available and that pertains to an unconventional enzymically inactive variant protein (Lovering *et al.*, 2011). Although this insightful structural study reveals possible roles of conserved residues in the enzyme activity of active domains, it is clear that the determination of the structure of RpfG and of the HD-GYP domain, both alone and particularly in combination with ‘target’ GGDEF domains, may offer information on the interacting surfaces and insight into the molecular basis of enzyme activity.

Although the interaction between RpfG and two GGDEF domain proteins is known to regulate motility, it is not known how protein–protein interactions between the HD-GYP : : GGDEF : : PilZ complex affect the activities of PilT and PilU, and whether they have influences on other bacterial functions. Equally, it is not known whether interaction with other GGDEF domain proteins occurs under different environmental or growth conditions, and whether these interactions serve to regulate different bacterial functions or processes. In addition, the biological relevance of HD-GYP domain interaction with other classes of bacterial regulatory protein that have been revealed by several Y2H analyses should be assessed.

The results of work that addresses these issues should contribute to our understanding of signal transduction pathways that involve the HD-GYP domain and cyclic di-GMP, not only in *Xcc* but also more widely in other pathogenic bacteria. Since HD-GYP domain proteins are known to regulate expression of virulence determinants or biofilm formation in several of these pathogens, elucidation of these pathways may identify key steps for interference with consequences for novel strategies for disease control.
